# Modeling suggests that microliter volumes of contaminated blood caused an outbreak of hepatitis C during computerized tomography

**DOI:** 10.1371/journal.pone.0210173

**Published:** 2019-01-15

**Authors:** Eyal Shteyer, Louis Shekhtman, Tal Zinger, Sheri Harari, Inna Gafanovich, Dana Wolf, Hefziba Ivgi, Rima Barsuk, Ilana Dery, Daniela Armoni, Mila Rivkin, Rahul Pipalia, Michal Cohen Eliav, Yizhak Skorochod, Gabriel S. Breuer, Ran Tur-kaspa, Yonit Weil Wiener, Adi Stern, Scott J. Cotler, Harel Dahari, Yoav Lurie

**Affiliations:** 1 Juliet Keidan Pediatric Gastroenterology Institute, Shaare Zedek Medical Center and the Hebrew University of Jerusalem, Jerusalem, Israel; 2 The Program for Experimental and Theoretical Modeling, Division of Hepatology, Department of Medicine, Loyola University Medical Center, Maywood, IL, United States of America; 3 Department of Physics, Bar-Ilan University, Ramat Gan, Israel; 4 Department of Molecular Microbiology and Biotechnology, School of Molecular Cell Biology and Biotechnology, Tel-Aviv University, Tel-Aviv, Israel; 5 Liver Unit, Shaare Zedek Medical Center and the Hebrew University of Jerusalem, Jerusalem, Israel; 6 Clinical Virology Unit, Hadassah Hebrew University Medical Center, Jerusalem, Israel; 7 Bar-Ilan University Faculty of Medicine in the Galilee, Safed, Israel; Centre de Recherche en Cancerologie de Lyon, FRANCE

## Abstract

**Background & aims:**

Acute hepatitis C (AHC) is not frequently identified because patients are usually asymptomatic, although may be recognized after iatrogenic exposures such as needle stick injuries, medical injection, and acupuncture. We describe an outbreak of AHC among 12 patients who received IV saline flush from a single multi-dose vial after intravenous contrast administration for a computerized tomography (CT) scan. The last patient to receive IV contrast with saline flush from a multi-dose vial at the clinic on the previous day was known to have chronic HCV genotype 1b (termed potential source, PS). Here we sought to confirm (via genetic analysis) the source of infection and to predict the minimal contaminating level of IV saline flush needed to transmit infectious virus to all patients.

**Methods:**

In order to confirm the source of infection, we sequenced the HCV E1E2 region in 7 CT patients, in PS, and in 2 control samples from unrelated patients also infected with HCV genotype 1b. A transmission probabilistic model was developed to predict the contamination volume of blood that would have been sufficient to transmit infectious virus to all patients.

**Results:**

Viral sequencing showed close clustering of the cases with the PS. The transmission probabilistic model predicted that contamination of the multi-dose saline vial with 0.6–8.7 microliters of blood would have been sufficient to transmit infectious virus to all patients.

**Conclusion:**

Analysis of this unique cohort provides a new understanding of HCV transmission with respect to contaminating volumes and viral titers.

## Introduction

Hepatitis C virus (HCV) continues to pose a major global public health problem including the burden of HCV-related cirrhosis and hepatocellular carcinoma [1]. The acute phase of HCV infection is usually defined as 6 months following initial infection [2]. Acute HCV (AHC) is not frequently identified because patients are usually asymptomatic, although may be recognized after iatrogenic exposures such as needle stick injuries, medical injection, and acupuncture [3–8]. Here we describe an outbreak of AHC, and establish a strong relationship between the predicted source of infection and the patients based on genetic sequencing and a phylogenetic analysis. Using these findings we applied probabilistic modeling to estimate the contamination level of blood necessary to cause the outbreak as a function of the viral load of the source.

## Methods

### HCV RNA extraction and reverse transcription

Nucleic acids were extracted from patient serum using the NucliSENS easyMAG kit (bioMérieux, Lyon, France). cDNA was synthesized with SuperScript III first strand (Thermo Fisher Scientific) using random hexamers and oligo-dTs, according to the manufacturer’s instructions. Notably, addition of RNaseOUT RNase inhibitor (Thermo Fisher Scientific) was found to be crucial. In order to maximize the number of viral genomes transcribed, we incubated the reverse transcriptase (RT) at 50°C for 2 hours prior to heat inactivation at 85°C for 5 minutes.

#### Amplification

The HCV E1 and E2 genes were amplified by nested PCR using Platinum SuperFi High Fidelity DNA Polymerase (Thermo-Fisher scientific). The first round of PCR was performed using 4 μL of RT reaction product and a set of external primers (5'-ACATGAAGCTTCGCCGACCTCATGGGGTACA -3', 5'-CTCCGGATATCGCAGCCATCTCCCGG TCCAT-3'). After an initial denaturation step of 98°c for 3 min, 13 cycles of 98°c for 30sec, 60°c for 30 sec and 72°c for 2 min. After cycling, a final extension period at 72°c for 5min was added. The second round of PCR was performed using 4μL of the first PCR reaction and a set of internal primers (5'-GTGAACTATGCAACAGGGAA-3', 5'-CAGAAGA ACACAAGGAAGGAGAG-3'). We used the same PCR protocol for both rounds, while running 27 cycles for the second round. After verifying the amplification success using gel electrophoresis, the nested PCR product was purified using AMPure XP system (Beckman Coulter Life Sciences) at 0.5X ratio. 60 μg of DNA was sequenced using Sanger sequencing (Hylabs). During sequencing, at each position the nucleotide presenting maximal signal peak is assigned. In some positions, signals with equivalent strengths were received for two bases (“double peaks”), thus prompting a random choice between them. This could have detrimental effects on the accuracy of phylogenetic inference, therefore such positions were omitted from analysis.

#### Interleukin-28B genotyping

Single-nucleotide polymorphisms (SNP) rs8099917 and rs12979860 in the Interleukin-28B gene (IL28B) were detected following PCR amplification and direct Sanger sequencing of the PCR product as previously described [9, 10].

#### Sequence analysis and phylogenetic tree creation

Patients and control sequences were aligned using webPRANK [11] and a phylogenetic tree was inferred using PhyML [12] with default parameters. One hundred bootstrap iterations were used to assess confidence of internal splits (branches).

### Mathematical modeling

To predict the probability, *p*_*tran*,_ of an IV saline flush event resulting in transmission we modified our recent HCV probabilistic transmission model [13] as follows (Eq 1):

ptrans=1−(1−q)n; n=VL*rb*s (Eq 1)

where *n* represents the number of HCV RNA IUs transmitted to the patient, q represents the infectivity to HCV RNA ratio, *VL* is the viral load in PS, *s* is the volume of PS contaminated blood in the mixture, r is the amount of solution transferred to the patient, and b is the volume of solution in the bottle at time of contamination with blood from PS.

#### Parameter estimations

For the *VL* we used the measured viral load of PS (2,919,153 IU/ml). Each patient received r = 2 ml of saline solution from the bottle in accordance with the CT protocol. We assumed a minimum of b~30 ml (since 12 patients received 2 ml), and a maximum of b~98 ml (if a new bottle was opened for PS). The saline was contaminated by an unknown amount *s* of blood from PS. We used a range for the parameter q, from q = 1:11 to *q = 1*:*100*. A higher q = 1:11 HCV RNA titer to infectivity ratio, was established from reverse titration studies in the chimpanzee model [14–17]. These data were all obtained using acute phase plasma prior to seroconversion. The presence of neutralizing antibodies to HCV in a plasma sample may have an impact on the proportion of transmitted virus that can result in an infection. Such antibodies are known to be present in sera from chronically infected patients [18, 19] (our predicted source, PS, was chronically HCV), and previous studies indicate that there is a continuous evolution of the virus such that it escapes the host immune response [20]. Thus, although circulating antibodies in PS may not fully functional against the transmitted virus, the presence of antibodies to HCV in PS could further reduce transmission probabilities and thus a lower q = 1:100 HCV RNA titer to infectivity ratio was assumed. Model simulations were performed using Python (version 2.7).

## Case reports

Initially three patients presented to Shaare Zedek Medical Center (SZMC) with new onset jaundice. Acute hepatitis C (AHC) was diagnosed, and patient history disclosed that all had a CT scan with IV contrast at the same clinic on March 17th 2016. The last patient to undergo a CT at the clinic on the previous day was known to have chronic HCV genotype 1b (referred to here as the predicted source, PS). Health authorities were notified, and all 12 patients who had undergone CT with contrast on the same day were summoned for testing. All 12 patients were found to have hepatitis C viremia. After thorough investigation, it was concluded that the viral source was a multi-use saline flush bottle that was used for each of the infected patients. All patients including the source patient were negative for Hepatitis B and HIV. All 12 AHC patients contracted HCV genotype 1b. Five patients were treated at another center [21]. The 7 patients who received care at SZMC provided the basis for this report. HCV RNA quantification and genotyping were assessed using the Abbott Realtime HCV assay (Abbott Laboratories, Abbott Park, IL, USA) and the Versant HCV genotype 2.0 assay (LiPA; Siemens Healthcare, Erlangen, Germany), respectively. Patient 5, who suffered from additional comorbidities, was diagnosed and treated for AHC at SZMC and then continued treatment for metastatic lung carcinoma and follow up for his hepatitis C at another hospital (see patient 6 in [21]). This study was approved by the local IRB committee of SZMC (148-18-SZMC).

**Table 1**depicts demographics, clinical features, and the indication for the CT scan. None of the patients had a history of elevated liver enzymes or fatty liver disease. Symptoms developed in 5 patients at a mean of 45±8.8 days after exposure and two patients remained asymptomatic. Weakness was prominent in all 5 symptomatic patients, nausea in 4 and 3 became jaundiced. Three patients (patients 5, 6 and 7) were negative for HCV antibody at time of diagnosis but became positive after 150, 92 and 84, days post infection (**Table 2**). These 3 patients had significantly (p>0.02) lower alanine aminotransferase (ALT) levels, i.e., 13±7 IU/L times the upper limit of normal (ULN), compared to the patients who were positive for HCV antibody (35±8 IU/L ULN). Three patients (patients 1, 3 and 4) cleared HCV RNA spontaneously, while 4 patients received DAA treatment (Fig 1).

**Fig 1:**
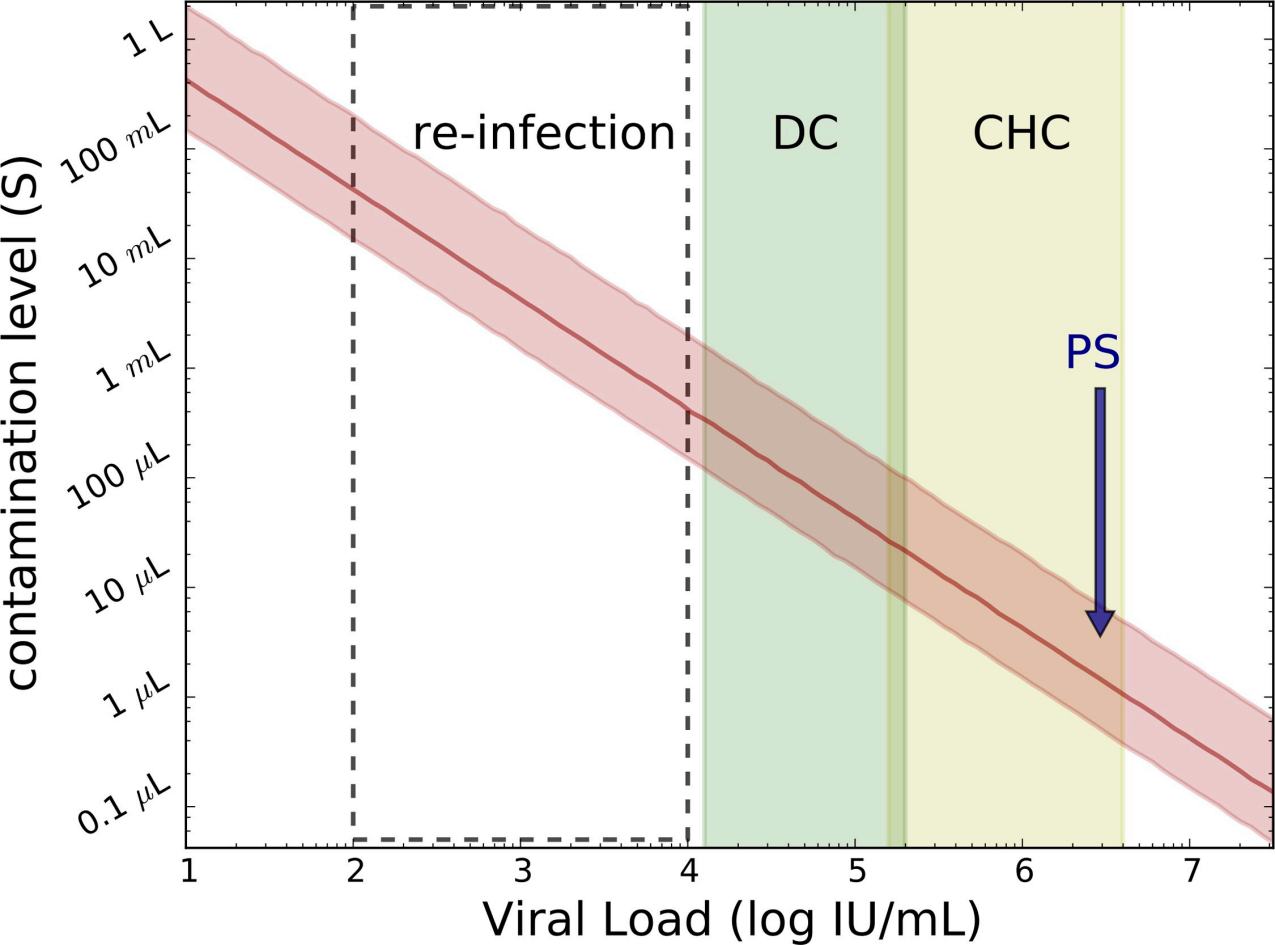
Viral load and ALT kinetics of seven AHC patients managed at SZMC. Open circles: observed HCV viral load above the limit of quantification, LOQ (>30 IU/mL); Red circles: observed HCV <LOQ but still detected; Green squares: observed HCV viral load below the limit of detection. HCV viral loads were assessed using the Abbott RealTime HCV assay (limit of quantification 30 IU/ml).

**Table 1:** Demographics, clinical features, and reason for CT scan.

Pt#	Gender	Age (years)	Medical History	CT indication	Symptomsat Presentation with HCV	Time to first symptoms (days)
**1**	F	31	None	Suspected lung mass	Weakness, nausea, vomiting & pruritus	
**2**	M	80	Cholelithiasis, hypertension, hypercholesterolemia, prostatic hypertrophy & diabetes mellitus	Headache	Weakness, nausea, anorexia & jaundice	45
**3**	F	16	backache	Back pain	Abdominal pain, anorexia, weakness, nausea & jaundice	45
**4**	F	25	none	Headache	Nausea, anorexia & malaise	46
**5**	M	72	COPD, lobectomy, hemodialysis, ischemic heart disease & hypothyroidism	Abdominal pain	Weakness	60
**6**	M	75	Diabetes mellitus	Renal stones	none	
**7**	M	71	Obesity & hypertension	flank pain	none	

**Table 2:** Viral load, serum biochemistries and IL-28B genotype in each patient.

**Pt#**	**HCV Abat diagnosis**	seroconvertion (days post exposure)	HCV RNA at diagnosis (IU/ml)	Total Bilirubin		ALTMax levels (Times ULN)		
				Presentation (mg/dL)	Max (mg/dL)		rs809917 genotype	rs12979860 genotype
1	Positive		3,749,887	16.2	24.1	32.69	TT	CC
2	Positive		283,904	13.5	24.5	42.49	GG	TT
3	Positive		2,629,902	4.2	5.5	43.25	TT	CT
4	Positive		8,230,152	1.3	1.8	23.28	TT	CC
5	Negative	150	14,661	0.6	0.6	3.07	GG	TT
6	Negative	94	7,414,650	1	1.7	21.8	TT	TT
7	Negative	82	817,310	0.6	1.5	14.8	TG	TT

Max- maximum. ULN, upper limit of normal; upper normal ALT levels 40 IU/L.

**Table 2**summarizes viral load, serum biochemistries and IL28B genotype in each patient. Median viral load at presentation was 2,629,902 IU/ml copies (range 14,661 to 8,230,152). Six patients had elevated bilirubin levels (range 1.5–24.5 mg/dl). ALT levels peaked 3 to 43 ULN.

**Fig 2:**
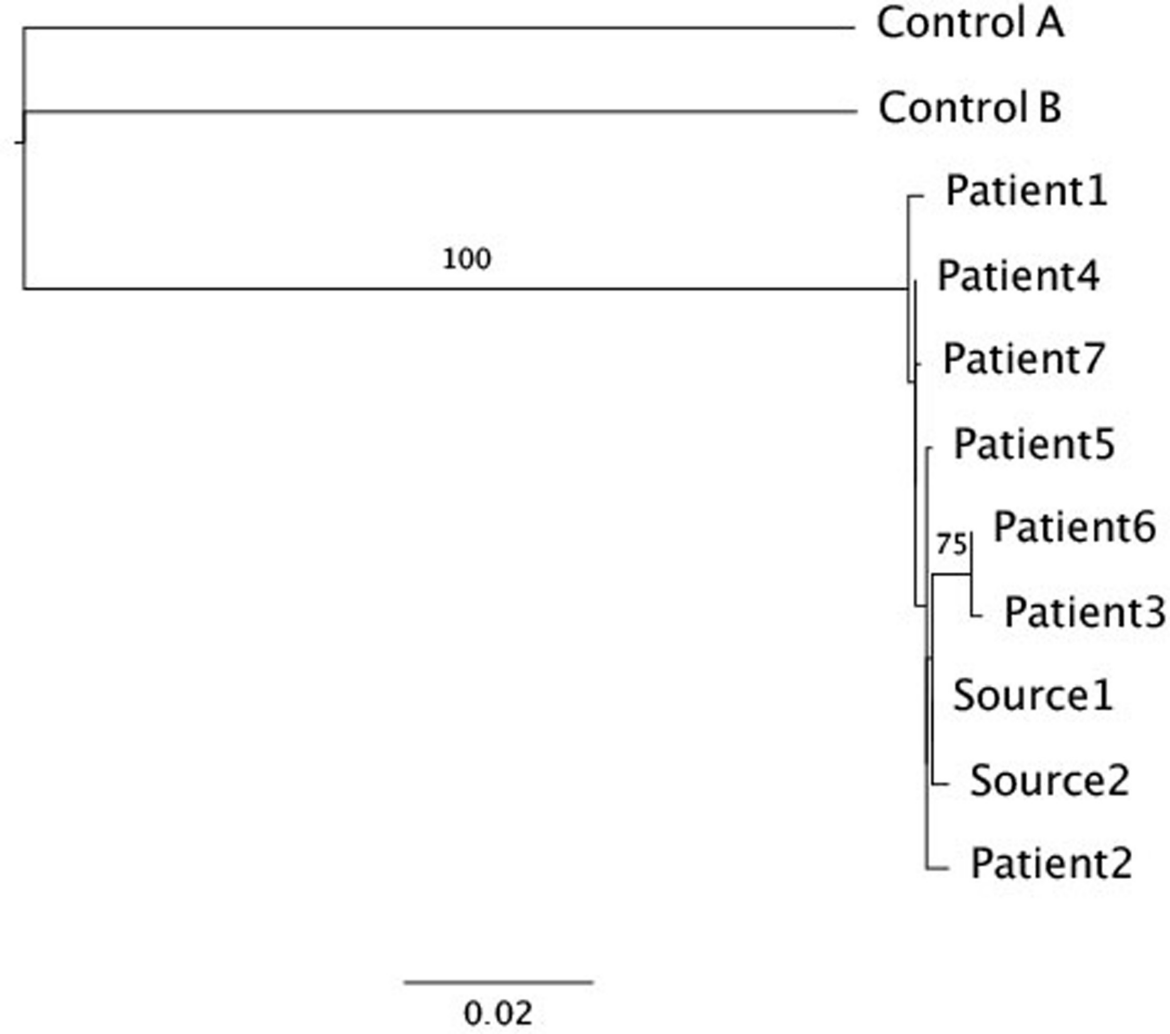
Phylogenetic tree based on maximum likelihood analysis of the E1-E2 region of the genome, with 100 bootstrap resampling iterations performed to assess confidence of the branches. Samples Control A and Control B are control samples. Samples Source1 and Source2 were taken from the predicted source of the infection. Samples Patient1-7 belong to the infected patients who were treated at SCMZ. A scale bar representing the expected number of substitutions per sites is shown at the bottom. Branch labels show bootstrap values higher than 70.

The PS had an HCV RNA level of 2,919,153 IU/ml and genotype 1b infection. Each AHC patient received 2 ml of saline flush from the same 100 ml saline bottle as the PS. In order to confirm the source of infection, we sequenced the entire E1E2 region of the genome (~1600 nucleotides) in 7 CT patients, in PS (two independent samples) and in 2 control samples from unrelated cases also infected with GT 1b. A phylogenetic tree showed tight clustering of sequences from PS and the 7 AHC cases, whereas the 2 control patients were found to be distant from the AHC cases and from each other (Fig 2). Strong bootstrap support (100%) separates all AHC patients and PS from the control sequences, providing evidence that PS was the source of the outbreak. Notably, inspection of the E1 and E2 sequences of the PS did not reveal any unique signatures of a more virulent strain.

**Fig 3:**
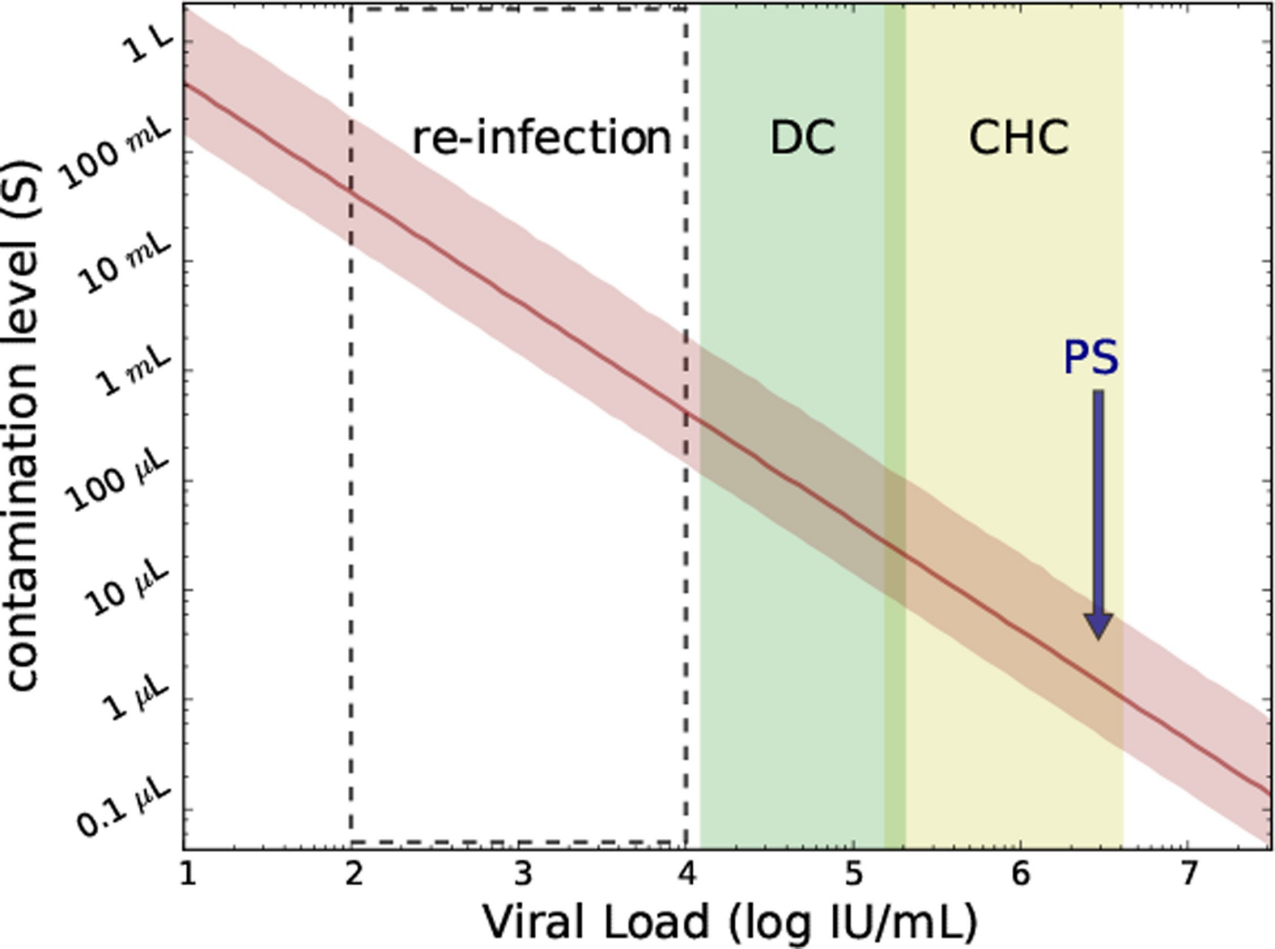
Predicting the contamination level (Eq 1; parameter s) needed for a 99.9% likelihood of infectious virus transmission in PS and *in-silico* subjects receiving contaminated IV saline flush during CT scan as a function of source viral load (median and 2.5%-97.5% percentiles are shown with red solid line and shaded red area, respectively). PS, viral load of predicted source; CHC, typical viral load range (mean±SD:5.9±0.7 IU/ml) in noncirrhotic chronic HCV-infected patients; DC, typical viral load (mean±SD:4.7±0.6) in decompensated cirrhotic HCV-infected patients; Re-infection, viral load in subjects with secondary infections, (approximately mean±SD:3±1 log IU/ml).

The model (Eq 1) was used to estimate the necessary contamination volume (parameter *s*) needed for a 99.9% likelihood of transmission of infection. Modeling projected a minimum contamination volume of the saline flush of 0.6–8.7 μl (or about 1 drop) of PS blood (Fig 3 and Table 3) would be sufficient to result in infection of all 12 patients.

**Table 3:** Projecting the necessary saline bottle contamination level based on individual’s infection.

Source type	VL mean+SD [log IU/ml]	Min *s* (95%CI) [μL]	Max *s* (95%CI) [μL]
**CHC**	5.9±0.7	1.7 (0.4–6.3)	43 (11–160)
**DC**	4.7±0.6	34 (8.7–130)	550 (140–2000)
**re-infection**	3.0±1.0	690 (180–2500)	6800 (1700–25000)

Projecting the necessary saline bottle contamination level ranges (median and 95% confidence interval, 95%CI) based on whether an individual’s infection (e.g. PS in the current study) is CHC (noncirrhotic chronic HCV), DC (decompensated cirrhotic HCV) or re-infected). The values used for these infection types are as described in the caption for Fig 3.

**Table 4**summarizes details regarding antiviral therapy. Three patients requested treatment despite recommendations for close observation to assess for spontaneous clearance. Only patient 7 started treatment per physician recommendation. Three patients received Ledipasvir/Sofosbuvir (patients 2, 6 & 7) and one was treated with Elbasvir/Grazoprevir (patient 5). It is of note that the two patients who were asymptomatic at presentation (patients 6 and 7) complained of weakness and fatigue after initiating HCV treatment. While untreated patients had viral fluctuations until viral eradication was achieved, all treated patients with DAAs experienced a rapid decline in viral load during the first 2 weeks post initiation of therapy (patients 2 and 5 became negative) followed by a 2^nd^ slower phase in patients 6 and 7 (Fig 1). In patient 6 viral load remained detectable until the end of therapy but was negative post treatment (as described in detail in [22]).

**Table 4:** Summary of patient management.

#Pt	Treatment	Reason for treatment	Drugs	Duration	Symptoms during follow up	Time to first negative HCV RNA (Days post exposure/ Day post initiation of treatment)
1	None	N/A	N/A	N/A	Weakness, nausea & abdominal pain	103 / N/A
2	Yes	Patient preference	Ledipasvir/ Sofosbuvir	12 weeks	Weakness, nausea & anorexia	145 / 14
3	None	N/A	N/A	N/A	Epigastric pain	112 / N/A
4	None	N/A	N/A	N/A	Weakness &Anorexia	166 / N/A
5	Yes	Patient preference	Elbasvir/ Grazoprevir	8 weeks	Weakness & anorexia	95 / 9
6	Yes	Patient preference	Ledipasvir/ Sofosbuvir	12 weeks	Anorexia	152 / 94
7	Yes	Persistence viral load	Ledipasvir/ Sofosbuvir	12 weeks	Weakness	159 / 91

N/A- not applicable.

## Discussion

Iatrogenic exposure is a risk factor for HCV transmission that can drive HCV outbreaks. We report here an unfortunate outbreak of HCV among a group of patients who were exposed to HCV while undergoing CT scans. Seven of the twelve patients infected in this outbreak were managed at our center (SMCZ) while five were managed at another center as recently reported [21]. Modeling of patient data (i.e., predicted source viral load) and features of the CT procedure that led to the outbreak (i.e., saline bottle volume range, volume of saline that each patient received and plausible range of HCV RNA titer to infectivity ratio) provided new insight into HCV transmission suggesting that very small amounts of contaminated blood (0.6–8.7 μl) diluted in saline from a CHC patient with a viral load within the typical viral load range in noncirrhotic chronic HCV-infected patients [23] can lead to infection of 100% of recipients. Higher contaminating volumes might be necessary for individuals with lower viral load such as decompensated cirrhotic patients [24] and those who have secondary infections [25], as shown in Fig 3.

Our probabilistic model (Eq 1) provides a more rigorous way to determine the minimum amount of HCV RNA needed to transmit infection compared to a previous estimate by Operskalski et al [26]. For HCV transmission via blood transfusion, Eq 1 can be simplified to P_trans_ = 1-(1-q)^n^ (termed modified model), where *q* parameter remains the RNA to infectivity ratio (same range 1:11 to 1:100 as in Eq 1 which is in agreement with the suggested range described by Operskalski et al) and *n* represents the total number of HCV RNA copies transfused to the recipient. Our probabilistic model predicts a high P_trans_ (99%) with a donor viral load as low as 1.6 copies per ml when 1 unit of packed red blood cells (PRBC), which generally includes 30 ml of plasma [26], is transfused. At the other extreme, the model projects a maximum low probability (P_trans_ = 1%) when *n* = 1 viral RNA copy is transmitted, i.e. if a donor viral load is lower than 0.033 IU/ml 1 unit of PRBC containing 30 ml of plasma transfused would contain <1 viral RNA copy.

The current study is novel compared to previous outbreak reports [27–29], in that it includes frequent longitudinal HCV RNA measurements post infection with and without DAA therapy and a probabilistic modeling approach. However, there are limitations inherent in the retrospective, real-world data-set. Five patients were not managed at SMCZ [21], precluding a comprehensive analysis of the outbreak. In spite of this, the detailed data obtained from the seven patients treated at SMCZ (and those managed elsewhere [21]) provided adequate information to confirm the source of the infection via phylogenetic analysis, and model the amount of HCV contaminated blood needed for transmission. Residual saline flush was not available for HCV RNA testing, although a careful evaluation of the CT protocol and the practices of the CT technicians identified the saline flush as the common source of exposure among the AHC patients rather than other routes such as deliberate infection as previously reported [8]. Lastly, since all but 3 patients were treated with DAA therapy shortly after diagnosis, a comprehensive immunological evaluation during AHC was not feasible. While the 3 patients who spontaneously cleared the virus (i.e., were not treated with DAA therapy) were female and had the favorable IL28B genotype (Table 2), which is in agreement with previous findings [30], though the present study does not inform which patients with AHC should be treated with DAA.

The current study highlights the importance of using extra care to ensure that no contamination occurs since even microliter amounts of infected blood diluted in saline can lead to HCV outbreaks, and further emphasizes the need for prevention strategies and vaccines to eliminate HCV transmission [31].

## Abbreviations

HCV: Hepatitis C virus
AHC: Acute Hepatitis C virus
CT: computerized tomography
PS: predicted source
DAA: direct-acting antiviral
