# Correction: Modeling suggests that microliter volumes of contaminated blood caused an outbreak of hepatitis C during computerized tomography

**DOI:** 10.1371/journal.pone.0212252

**Published:** 2019-02-07

**Authors:** Eyal Shteyer, Louis Shekhtman, Tal Zinger, Sheri Harari, Inna Gafanovich, Dana Wolf, Hefziba Ivgi, Rima Barsuk, Ilana Dery, Daniela Armoni, Mila Rivkin, Rahul Pipalia, Michal Cohen Eliav, Yizhak Skorochod, Gabriel S. Breuer, Ran Tur-kaspa, Yonit Weil Wiener, Adi Stern, Scott J. Cotler, Harel Dahari, Yoav Lurie

The image for Fig 1 is incorrectly a duplicate of the image for Fig 3. Please view the correct Fig 1 below:

**Fig 1:**
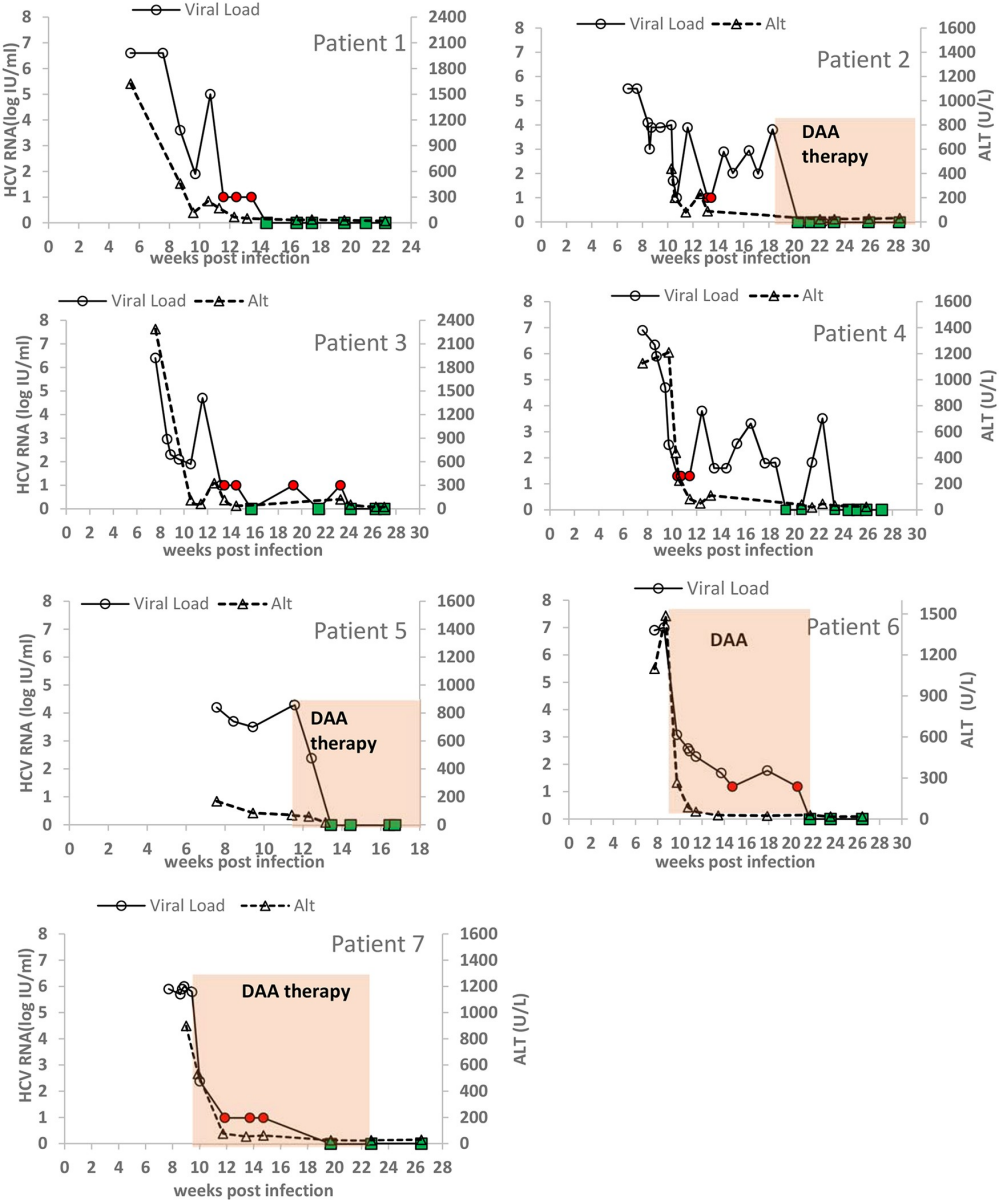
Viral load and ALT kinetics of seven AHC patients managed at SZMC. Open circles: observed HCV viral load above the limit of quantification, LOQ (>30 IU/mL); Red circles: observed HCV <LOQ but still detected; Green squares: observed HCV viral load below the limit of detection. HCV viral loads were assessed using the Abbott RealTime HCV assay (limit of quantification 30 IU/ml).

There are errors in the Author Contributions. The correct contributions are: Conceptualization: ES, HD, YL. Data curation: ES, IG, DW, ID, DA, MCE, YS, GSB, HD, YL. Formal analysis: LS, HI, RB, RP, RTK, YWW, AS, SJC, YL. Investigation: TZ, SH, MR. Methodology: LS, TZ, SH, RB, HD. Supervision: ES, HD. Writing—original draft: ES, LS, TZ, HD. Writing—review & editing: ES, DW, AS, SJC, HD.

The following information is missing from the Funding section: This study was supported in part by a fellowship to TZ from the Edmond J. Safra Center for Bioinformatics at Tel-Aviv University.
